# Current State of the Art of Newborn Screening for Lysosomal Storage Disorders

**DOI:** 10.3390/ijns4030024

**Published:** 2018-07-18

**Authors:** David S. Millington, Deeksha S. Bali

**Affiliations:** Department of PediatricsDivision of Medical Genetics, Duke University School of Medicine, Durham, NC 27709, USA

**Keywords:** lysosomal storage disorders, digital microfluidics, tandem mass spectrometry, fluorometry, enzyme assays, newborn screening results

## Abstract

Prospective full-population newborn screening for multiple lysosomal storage disorders (LSDs) is currently practiced in a few NBS programs, and several others are actively pursuing this course of action. Two platforms suitable for multiple LSD screening—tandem mass spectrometry (MS/MS) and digital microfluidic fluorometry (DMF)—are now commercially available with reagent kits. In this article, we review the methods currently used for prospective NBS for LSDs and objectively compare their workflows and the results from two programs in the United States that screen for the same four LSDs, one using MS/MS and the other DMF. The results show that the DMF platform workflow is simpler and generates results faster than MS/MS, enabling results reporting on the same day as specimen analysis. Furthermore, the performance metrics for both platforms while not identical, are broadly similar and do not indicate the superior performance of one method over the other. Results show a preponderance of inconclusive results for Pompe and Fabry diseases and for Hurler syndrome, due to genetic heterogeneity and other factors that can lead to low enzyme activities, regardless of the screening method. We conclude that either platform is a good choice but caution that post-analytical tools will need to be applied to improve the positive predictive value for these conditions.

## 1. Introduction

Newborn screening (NBS) for inborn errors of metabolism (IEM) began over 50 years ago with Robert Guthrie’s bacterial inhibition test to detect phenylketonuria by means of elevated phenylalanine concentrations in dried blood spots collected during the first few days of life [1]. During the ensuing period covering more than 30 years, a few additional tests for conditions that satisfied criteria published by Wilson and Jungner in 1968 [2] were added to the panel. For the most part, by the mid-1990s, most programs in the United States were screening for only a few conditions, which included phenylketonuria, congenital hypothyroidism, congenital adrenal hyperplasia and galactosemia. At that time, the concept of multiplex testing was introduced using tandem mass spectrometry (MS/MS) [3,4]—a method that detects multiple acylcarnitines and amino acids simultaneously and recognizes more than 30 IEM [5,6,7]. The widespread adoption of this practice over the subsequent decades has dramatically changed the landscape of NBS. Initially, there was confusion and concern regarding the number of conditions that multiple analyte testing by MS/MS was potentially able to detect. Furthermore, many of the conditions detectable by MS/MS do not satisfy the Wilson and Jungner criteria. In an attempt to bring some order to this somewhat chaotic situation, the Health Resources and Services Administration (HRSA), an agency of the U.S. Department of Health and Human Services, requested assistance from the American College of Medical Genetics (ACMG). The ACMG formed a Committee to conduct a systematic evaluation of newborn screening programs and reviewed over 80 conditions for possible inclusion in a universal recommended panel that NBS programs could regard as a standard. The result was a report that recommended 29 primary targets (the “core disorders”) for universal screening, subsequently referred to as the ‘recommended uniform screening panel’ (RUSP) [8]. The same report identified a further 26 conditions, referred to as secondary conditions, that expanded NBS may detect in addition to those on the RUSP [8,9]. The ACMG Committee established more stringent criteria for inclusion of conditions to the RUSP and was later encompassed by the U.S. Department of Health and Human Services Secretary’s Advisory Committee on Heritable Disorders in Newborns and Children (SACHDNC) [10]. This committee has subsequently recommended the inclusion of severe combined immune deficiency (SCID), Critical Congenital Heart Defects (CCHD), and most recently Pompe Disease (Glycogen Storage Disease-II), Hurler Syndrome (MPS I) and X-linked adrenoleukodystrophy (X-ALD) to the RUSP, making a total of 34 core conditions as of the time of writing.

The inclusion of Pompe and Hurler, both of which are examples of lysosomal storage disorders (LSDs), to the RUSP is proving to be challenging and controversial [11,12]. Part of the driving force for the inclusion of LSDs is the development of promising new treatment options such as enzyme replacement therapy and hematopoietic stem cell transplantation, as well as the development of new screening tests based on the measurement of enzymatic activity [13,14]. Another contributing factor has been the advocacy of special interest groups that have successfully lobbied the legislators in certain state programs in the US to mandate screening for LSDs regardless of the SACHDNC procedures and recommendations. Thus, New York was compelled to screen for Krabbe disease [15], while Illinois and Missouri issued mandates to screen for Krabbe and five additional LSDs prior to the addition of any LSDs to the RUSP [16,17].

Programs that are currently on the cusp of screening for at least the two LSDs currently on the RUSP (Pompe and Hurler disorders) are faced with a choice between the few viable methods that are currently in use in NBS programs. The purpose of this review is to compare objectively the options that are applicable to high throughput measurement of multiple enzymes for the detection of LSDs, based on currently available and published data.

## 2. Materials and Methods

### 2.1. Introduction

Newborn screening programs currently have three options available for high-throughput screening of multiple LSDs that use commercially available synthetic substrates to quantify enzyme activity in newborn dried blood spot samples. A method for multiplex immunoassay of LSD proteins that takes advantage of micro-bead array technology has been developed and applied retrospectively for NBS [18]. Despite showing promise, it does not appear to have been further developed for prospective NBS, perhaps owing to the lack of commercially available reagents.

### 2.2. Microtiter-Plate Fluorometry

This option requires the application of a benchtop microfluorometer that accepts 96-well microtiter plates to measure the release of the fluorophore 4-methylumbelliferone (4-MU) from a synthetic substrate targeted to a specific LSD enzyme in a DBS extract after an 18 h incubation. This method, which is based on the pioneering work of Chamoles and co-workers [19,20,21], has been used successfully in Taiwan to screen for both Pompe [22] and Fabry [23] disease and has the advantages of simplicity and low operational expense, as well as performing the assays under optimal conditions that include specific pH, additives and buffers. Because new reagents are now available commercially that have improved the performance of fluorometric assays, this method represents a viable and cost-effective option for programs that wish to screen for only one or two LSDs. Limitations include the fact that in the current iteration of this method, each assay requires a separate DBS punch, thus it is unsuitable for expansion to multiple LSD screening. Another drawback is the delay in time to report screening results resulting from the lengthy incubation time. A presumptive positive result would prompt a re-analysis in duplicate of the same DBS, requiring an additional 18 h incubation with a calculation of the mean enzyme activity prior to result reporting.

### 2.3. Digital Microfluidic Fluorometry

The digital microfluidics (DMF) platform for LSD newborn screening was originally developed by the Advanced Liquid Logic Company (now Baebies, Inc., Durham, NC, USA) to simultaneously perform up to 5 fluorometric LSD enzyme assays using a single DBS punch [24,25]. Each automated enzyme reaction takes place in a discrete droplet on a disposable cartridge that incorporates reservoirs for 5 reagents and 48 samples. Typically, four of the sample reservoirs are charged with 4-MU calibrators, leaving the others to accept up to 40 patient samples and 4 quality controls. Unlike true multiplexing (where multiple reactions are performed in the same environment), the “spatial multiplexing” DMF method permits each LSD enzyme reaction to be performed in an individual droplet under its individually optimized conditions (pH, inhibitors, buffer). DMF fluorometry also provides notable improvements over the microtiter plate fluorometry methods such as improved reagent purity, much shorter reaction time (~2 h) and lower cost (due to the nanoliter reaction volumes and subsequent reagent savings). Importantly, this method also has the unique advantage of virtually limitless capacity to add further assays for LSDs and other conditions without requiring more sample punches while exploiting the “spatial multiplexing” to independently add new assays. This platform offers the fastest method to screen LSDs among the currently available methods [26], with turnaround times similar to those of tandem mass spectrometry NBS for acylcarnitines and amino acids. Repeat testing of specimens with presumptive positive results can be accomplished within a few hours, thus enabling same-day result reporting.

The first version of DMF platform was initially used prospectively for LSD newborn screening in 2010 for a pilot study in the Illinois NBS program, when over 8000 specimens were screened for 3 LSDs—Pompe, Fabry, Gaucher [27]. This DMF program was suspended because of low throughput (only 12 samples per cartridge at the time) and lack of reagents for the assay to detect Krabbe disease. Nevertheless, this limited pilot did identify confirmed patients with Gaucher (2) and Fabry (7) disease [27]. The Missouri NBS program began using a more advanced version of the DMF platform in January 2013 that analyzes 44 samples per cartridge for the same 4 LSDs [16]. Their program has been operating on a continuous basis without modification since then, has already screened over 300,000 babies, and has detected more than 130 LSD cases confirmed through diagnostic testing [28]. This program therefore represents the longest continuously active prospective multiple LSD screening program in the United States that is still ongoing. Further implementation and use of the DMF SEEKER^®^ platform in LSD screening was limited due to the delays in obtaining approval from the U.S. FDA for its use as a medical device. Such approval was granted in early 2017 [29] for the platform and reagent kits and thus there is no impediment to its future deployment in other NBS programs for LSD screening. In addition to Missouri, DMF instruments are now installed and being actively used in the Michigan public health laboratory and are undergoing validation in the Maryland public health laboratory.

### 2.4. Tandem Mass Spectrometry

Tandem mass spectrometry (MS/MS) has been widely used in NBS programs worldwide for over 20 years to screen for disorders of fatty acid oxidation, branched-chain amino acid catabolism and aminoacidopathies. As is the case with the fluorometry protocols, MS/MS based NBS for LSD assesses the risk for enzyme deficiency in DBS extracts using specific enzyme activity assays. The first example was an assay for galactocerebrosidase (GALC) deficiency, published by Gelb and associates in 2004 [30]. This method was adopted by the state of New York for their pilot program to screen for Krabbe disease [31]. There soon followed a “multiplex” assay for 5 LSD enzymes by MS/MS [32]. This method requires, for each enzyme of interest, an initial reaction with an artificial substrate specific to that enzyme, plus an additional reagent to quantify the product of the reaction by means of MS/MS (selected reaction monitoring). Initially, the method required separate DBS punches and reaction wells for each enzyme, a 24-h incubation time, followed by a cumbersome additional work-up prior to the analysis of all the reaction products by MS/MS. A succession of modifications to the multiple LSD screening protocol has been published since then that have overcome many of these limitations. It is beyond the scope of this article to review all of the numerous protocols that have been utilized in various pilot NBS studies for LSDs involving MS/MS; the reader is referred to a recent review in this Journal [33]. Current protocols for multiple LSD enzyme assays suitable for NBS now use a single DBS punch and a single reaction well or tube, from which all reaction products are analyzed simultaneously by MS/MS [34]. These reactions are conducted in an aqueous environment that necessitates either a solid-phase and/or liquid-liquid extraction to eliminate contaminants before injection into the mass spectrometer, or the addition of an ultra-performance liquid chromatography (UPLC) column in-line with the MS/MS to accomplish the same purpose [34,35].

The state of Illinois represents arguably the most pertinent example of the current state-of-the-art in multiple LSD newborn screening by MS/MS. In a recent article, the program summarized its initial 15-month experience from 1 November 2014 to 31 August 2016 [17], during which time they screened almost 220,000 babies for five LSDs (Fabry, Hurler, Gaucher, Pompe, and Niemann-Pick A/B). Their method uses a commercially available reagent kit that contains buffer, substrates and internal standards for the 5 enzymes, plus inhibitors of non-LSD enzymes that compete for the substrates for Pompe, Fabry and Hurler. It is noteworthy that the incubation time is 17 h, essentially the same as that required by the standard benchtop fluorometric method, perhaps because the single reaction protocol inherently compromises the assay conditions for several enzymes by using a fixed pH (4.7), whereas the optimal pH for these enzyme reactions ranges from 3.5 to 5.2 [24]. The authors also used in-line UPLC to avoid the phase separation step and keep the MS/MS analyzer free of contaminants, thus enabling five 96-well plates to be analyzed on the same instrument within a 24-h period. This protocol is, however, incompatible with the current methods in use for analysis of acylcarnitines and amino acids by MS/MS, which uses direct flow-injection from 96-well plates. Thus, it is important to recognize that for this and other logistical reasons, programs intending to deploy MS/MS to screen for LSDs will need to invest in significant additional resources, including tandem mass spectrometers with UPLC systems, the infrastructure to support them, additional trained technologists, plus ongoing service and maintenance costs [36]. State programs using MS/MS for LSD screening currently include New York, Illinois, Tennessee, Kentucky, Massachusetts, Minnesota and Ohio.

### 2.5. Workflows

**Figure 1:**
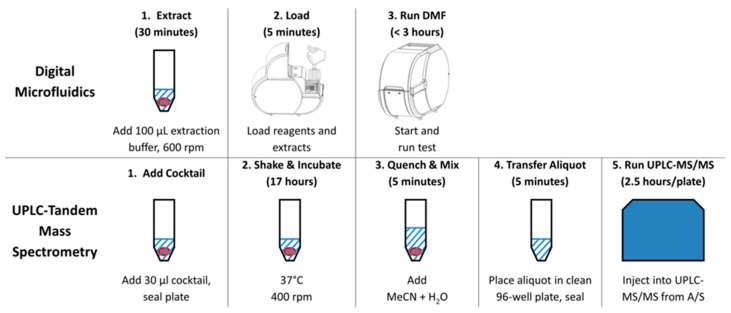
Comparison of workflows for multiple LSD newborn screening using the digital microfluidic and ultra-performance liquid chromatography-tandem mass spectrometric platforms. The red circles in the picture above depict a single blood spot punch from DBS card. These workflows are extracted from the methods published by the Missouri NBS program using DMF [16] and the Illinois NBS program using UPLC-MS/MS [17].

Workflows for the methods currently applicable to multiple LSD screening are compared in Figure 1. The workflow for DMF is that utilized by the Missouri NBS program since the inception of LSD screening in 2013 [16], and is standard for this method. The workflow for UPLC-MS/MS is the method published by the Illinois NBS program in their most recent article [17]. Although numerous alternative workflows for LSD NBS by MS/MS have been published elsewhere [33], the Illinois protocol is arguably the least complex and most suitable for high-throughput NBS. The DMF workflow is obviously the more straightforward of the two, and may be particularly attractive for developing programs that do not have access to MS/MS.

## 3. Results

In this section, we review published results from programs that have ongoing, full population screening for multiple LSDs.

### 3.1. Results from the Taiwan Program

Taiwan has the longest continuous prospective screening program for LSDs. Screening for Pompe disease using a fluorometric assay using a 96-well plate reader for alpha-glucosidase began in 2005, initially in about half of the population, using the non-screened population as controls. After the first 18 months, about the same number of cases of the severe, infantile form of the disease (IOPD) was detected in both populations, but the early identification of the screened cases resulted in earlier diagnosis and treatment, with the expectation of better outcomes than those in the control group [22]. The screening program was deemed to be effective and expanded to the full population. By the end of 2011, over 470,000 babies were screened, resulting in nine IOPD newborns diagnosed and receiving their first enzyme replacement therapy (ERT) prior to the age of 1 month [37]. The screening program detected later onset cases of Pompe disease (LOPD) that were also shown to benefit from early diagnosis, as well as numerous gene variants with pseudodeficiency alleles [38,39]. Taiwan also began screening for Fabry disease [40] using the same platform with a modified version of the previously reported method [20]. The Taiwan program later switched from the 96-well fluorometry platform to MS/MS for its full population screening for four LSDs including Pompe and Fabry disease [41,42]. The authors compared results for Pompe and Fabry between the two platforms and concluded that MS/MS outperformed fluorometry with noticeable improvements in PPV for these conditions. The reagents used for fluorometric assays in Taiwan cannot, however, be sourced commercially and results therefore may not be comparable with those obtained by DMF fluorometry. Regardless, there are numerous reports claiming superior performance for MS/MS relative to fluorometry, and by extension to DMF for LSD screening [33,43,44,45]. These conclusions are largely based on retrospective studies, not prospective screening, and challenges to them have led to a spirited exchange of views between experts in the field [36,46,47,48]. Prospective data from newborn screening programs in the U.S. are now available for critical comparison.

### 3.2. Results from the Missouri and Illinois State Programs

LSD newborn screening algorithms in the U.S. vary widely between state programs, with each state selecting different combinations of LSDs, also applying its own brand and combination of second-tier enzyme testing, second-tier molecular analysis and/or post-analytical tools [49]. Such differences make it difficult to compare screening results obtained in different programs. At the time of writing, only two programs in the U.S. have published results from ongoing, prospective newborn screening for the same multiple LSDs; Missouri using DMF and Illinois using UPLC MS/MS. The screening algorithms by these programs have thus far been quite similar, with no second tier or post-analytical tools applied, and the results therefore represent an analogous comparison of the two different screening methods. The calculated incidences for Pompe, Hurler, Fabry and Gaucher in each state (Table 1) align well with previously published incidence rates. There are, however, some notable differences between programs with Missouri reporting greater than 2-fold higher incidences of Pompe and Fabry. It is possible that differences in ethnicity and case definition criteria may account for at least some of these differences.

In terms of assay performance, positive predictive values (PPV) reported for the various conditions ranged from 2% (for Hurler) to 59% (for Fabry) in Missouri and from 1% (for Hurler) to 31% (for Fabry) in Illinois. Missouri reported generally higher PPV and lower false positive rates compared with Illinois for all 4 LSDs; however, it is expected that the Illinois program will see improved PPV and fewer false positives as they fine-tune their cut-off values. Also of note, the PPV for Pompe in Missouri decreased in their 4 year data set [28] relative to results published after just 6 months of screening [16]. This change is attributed primarily to the larger number of samples (approximately 6-fold more) included in the 4-year results relative to the original 6-month results and underscores concerns raised by others regarding the challenges inherent to rare disease research using relatively small sample sizes [52].

## 4. Discussion

The prospective screening data from Illinois and Missouri (Table 2) do not support previous reports claiming superior performance and lower false positive rates for MS/MS relative to DMF for LSD screening, which, as stated earlier, are based largely on retrospective studies. Rather, these results highlight the fact that either method can be used for efficient LSD newborn screening. The results show that pseudo-deficiency alleles that exhibit low enzyme activity in an individual with no disease phenotype and normal endogenous enzyme function, contribute to higher screen positive rates, higher false positive rates and have complicated the implementation of LSD NBS, especially for Pompe and Hurler. Further complications arise from the detection of high rates of later onset forms of Pompe, Hurler and Fabry disease. These issues are clearly independent of the screening method, as others have observed [26].

The practice of LSD screening, currently predicated on cut-offs for low enzymatic activity and second-tier molecular testing, may need to be altered to take account of the problems generated for health providers and families by inconclusive results [17]. Minter Baerg, et al. [49] make a strong argument in favor of incorporating multivariate pattern recognition software (Collaborative Laboratory Integrated Reports (CLIR; https://clir.mayo.edu)) into the NBS algorithm, a post-analytical tool that can dramatically reduce false positive outcomes. This tool requires input of demographic data, such as time of specimen collection and gestational age, and at least three enzyme activity values from the same specimen in order to be effective. Second-tier biochemical testing for some LSDs has recently become available and may also be effective, especially in identifying patients at risk for neonatal onset forms of Pompe [53] and Hurler [54].

Newborn screening for LSDs began slowly in the US, but is now in a phase of steady expansion. The incorporation of post-analytical tools to increase the positive predictive value of the LSD primary screening tests, especially for Pompe, Hurler and Fabry, is an obvious quality improvement that is urgently needed, and is likely to become more prevalent in the future. Evidence from the literature thus far supports this view and will enable screening programs to defer molecular testing until after the majority of false positives have been eliminated by these other means. In anticipation of the addition of more LSDs and perhaps other conditions requiring enzymatic assays to the RUSP, multiplexed MS/MS protocols for as many as 22 disorders have been described [55], but require multiple DBS punches. Preliminary reports also indicate that DMF technology can support simultaneous testing of up to 10 different disorders from the extract of a single DBS punch [56].

We conclude that newborn screening for multiple LSDs can be successfully accomplished with essentially equivalent outcomes, by either MS/MS or DMF. The workflow for DMF is, however, simpler than that of MS/MS and facilitates same-day screening and reporting of critical results. Both methods are expandable to enable screening for additional conditions, if required. LSDs are clinically heterogeneous disorders that present as a spectrum of disease severity, and screening for them, regardless of the method used, identifies cases with reduced enzyme activity due to pseudodeficiency, later onset variants and other factors that lead to higher screen positive rates than other NBS conditions. As mentioned in the foregoing paragraph, new post-analytical tools, such as Collaborative Laboratory Integrated Reports (CLIR; https://clir.mayo.edu)) [49] and second-tier biochemical testing [53,54] are becoming available to address these issues.

## Figures and Tables

**Table 1:** Comparison of LSD incidence based on literature review with prospective screening results from Missouri and Illinois.

Disorder	Published Incidence	Incidence MO [16]	Incidence IL [17]
Pompe	1:28,000 [50]	1:9625	1:22,000
Hurler	1:87,000–1:185,000 [51]	1:154,000	1:220,000
Fabry	1:1500–1:13,000 [26]	1:3277	1:8500
Gaucher	1:57,000 [26]	1:61,600	1:44,000

**Table 2:** Performance criteria of LSD newborn screening assays in the Missouri and Illinois public health laboratories.

	GAA (Pompe)		IDUA (MPS I)		GLA (Fabry)		GBA (Gaucher)	
	MO (DMF)	IL (MS/MS)	MO (DMF)	IL (MS/MS)	MO (DMF)	IL (MS/MS)	MO (DMF)	IL (MS/MS)
Total infants	308,000	219,793	308,000	219,793	308,000	219,793	308,000	219,793
Screen Positives	**161**	**139**	**133**	**151**	**179**	**107**	**37**	**117**
Pending	0	8	0	24	0	16	0	19
Refused/Lost to f.u./Died	2	n/a	5	n/a	12	n/a	2	n/a
Normal	48	87	45	87	66	59	22	91
Carrier	39	15	8	5	0	0	5	0
Pseudodeficient	31	15	63	30	0	16	0	0
Undetermined /VUS/GUS ^3^	9	4	2	4	6	6	2	2
Confirmed Positive	**32**	**10**	**2**	**1**	**94**	**10**	**5**	**5**
PPV ^1^	26%	11%	3%	4%	60%	18%	21%	7%
False Positive rate ^2^	0.04%	0.05%	0.04%	0.06%	0.02%	0.03%	0.01%	0.04%
**False positives per 100,000**	38.3	53.2	37.7	55.5	21.4	34.1	8.8	41.4

^1^ Positive predictive value (PPV) calculated as [confirmed pos + VUS]/[screen pos + lost + refused + died]; ^2^ False positive rate (FPR) calculated as: normal + carrier + pseudo/total; ^3^ Undetermined/VUS/GUS includes samples with 1 pathogenic mutation + 1 VUS, 2 VUSs, or single VUS for Fabry. VUS: Variant of unknown significance; GUS: Genotype of unknown significance or onset.
